# The effect of Coronavirus disease pandemic on maternal and neonatal health: A cohort study from Isfahan, Iran

**DOI:** 10.18502/ijrm.v21i2.12806

**Published:** 2023-03-08

**Authors:** Hourieh Ansari, Zahra Amini, Elham Madreseh

**Affiliations:** ^1^Department of Community and Family Medicine, School of Medicine, Isfahan University of Medical Sciences, Isfahan, Iran.; ^2^Department of Epidemiology and Biostatistics, School of Health, Tehran University of Medical Sciences, Tehran, Iran.; ^3^Rheumatology Research Center, Tehran University of Medical Sciences, Tehran, Iran.

**Keywords:** Maternal health, Neonatal health, Pregnancy, COVID-19.

## Abstract

**Background:**

The Coronavirus disease-2019 (COVID-19) pandemic may profoundly impact on maternal and neonatal health worldwide. However, a few studies have investigated this topic.

**Objective:**

This study aimed to investigate the impact of the COVID-19 pandemic on maternal and neonatal health.

**Materials and Methods:**

This retrospective cohort study analyzed collected data from March to May 2020, and the same period in 2019, involving 5711 pregnant women referring to comprehensive healthcare centers in Isfahan province health facilities, Iran. Pregnant women and neonates were followed-up until 40 days after the delivery. Demographic characteristics, pre-pregnancy, antenatal care, and post-pregnancy variables were collected.

**Results:**

A total of 5711 pregnant women were studied, of whom 3477 (61%) were referred in 2019 (before the COVID-19 pandemic as nonexposed) and 2234 (39%) during the COVID-19 pandemic (as exposed group) in 2020. For those living in cities with a population of > 20,000, the number of antenatal care were lower about 2% compared to nonexposed group (p = 0.01). The number of mothers with a history of the underlying disease who referred to a comprehensive healthcare center during the COVID-19 pandemic (47%) was lower about 6% compared to nonexposed group (41%) (p < 0.001). During the COVID-19 pandemic, the prevalence of hypertension and gestational diabetes mellitus was 5% (n = 109) and 20% (n = 445), which were higher about 2% and 4%, respectively, compared to nonexposed group. The COVID-19 pandemic had no other significant effect on mothers' and neonates' other characteristics than nonexposed group.

**Conclusion:**

The COVID-19 pandemic imposes no significant effect on mothers' and neonates' health compared to nonexposed group.

## 1. Introduction

Coronavirus disease-2019 (COVID-19), which first broke out in Wuhan, China at the end of 2019, has rapidly spread worldwide. Though with a lower fatality rate, the extent of the pandemic (e.g., infected patients) has far exceeded previous outbreaks of severe acute respiratory syndromes and the middle east respiratory syndrome (1). The impact of COVID-19 varies from country to country, with a major contribution to the resilience levels of health systems as well as pandemic-control policies on public health infrastructure, societies, and the global economy (2). The pandemic has posed a huge challenge to health systems and has led to unprecedented challenges to public health, food systems, and the world of work, among various fields (3). Hence, an intertwining among health system-related parameters, policies on pandemic control, and socioeconomic factors have resulted in several negative consequences, including disrupted provision of healthcare services, increased poverty, and poor nutrition. In this line, there are concerns that the pandemic disproportionately burdened vulnerable groups, even in countries with universal health coverage. Even before the pandemic, there were in-depth health inequalities and problems in accessing healthcare services has intensified the concerns (4).

Similar to several health systems, the Iranian health system has prioritized provisions of healthcare services and temporarily suspended elective procedures, leading to a disrupted continuum of care. Reproductive health is one of the most important fields that probably has been affected by this policy. For instance, on the one hand, the number of recommended antenatal follow-up sessions has declined based on the policy to control the pandemic; on the other hand, as pregnancy is associated with significant physiologic and immunologic changes to support the fetus, pregnant women are at increased risk of various infections, including COVID-19, which declined their inclination to refer to healthcare facilities (5). “As evidenced by a survey conducted by the World Health Organization, 53% of the 105 participating countries reported partial disruptions in antenatal care and 32% in facility-based delivery services during the first several months of the COVID-19 pandemic” (6).

Noteworthy, the impact of the COVID-19 pandemic on maternal and neonatal health is not limited to the morbidity and mortality caused directly by the disease itself (7).

In this line, this study aimed to evaluate the effect of the COVID-19 pandemic on maternal and neonatal health among pregnant women referring to comprehensive healthcare centers in Isfahan province of Iran.

## 2. Materials and Methods

### Study design

Following a retrospective cohort design, data of all pregnant women whose last menstrual period dates were from March to May, 2019 (before the pandemic as nonexposed group) and referred to an urban or rural comprehensive healthcare center in Isfahan province, Iran (n = 3477) and all those who referred to these centers during this period in 2020 (during the pandemic as exposed group) (n = 2234), were surveyed. Required data were extracted from the electronic health records of participants.

### Study variables

Data on the following variables were collected:

• Demographic characteristics, including age and place of residence (i.e., cities > 20,000 population, cities < 20,000 population, villages, and marginalized areas);

• Pre-pregnancy variables, including weight, history of abortion, number of previous pregnancies, and history of underlying disease;

• Antenatal care, including the number of antenatal consultations, risky symptoms during pregnancy (e.g., hypertension, gestational diabetes, dyspnea, edema, and hemorrhage); and

• Post-pregnancy variables include height, weight, head circumference, type of delivery, and neonatal status (alive or stillbirth).

Pregnant women were followed up to 40 days after birth. According to the protocol of the Ministry of Health, Treatment and Medical Education for the standardization of maternal care, low-risk pregnant women should receive a total of 8 antenatal care consultations: 2 consultations in the first half of pregnancy (6 ${}^{\text{th}}$ -20 ${}^{\text{th}}$ wk) and 6 consultations in the second half (21 ${}^{\text{st}}$ -40 ${}^{\text{th}}$ wk) (8). However, since the onset of the COVID-19 pandemic, the total number of consultations has been reduced to 4 for low-risk women (i.e., those with no chronic disease and no COVID-19 infection). So, the first consultation should be provided during the first 6-10 wk of pregnancy, the second one should be provided during 24-28 wk of pregnancy, the third one should be provided during 31-34 wk of pregnancy, and the last one should be around 37 wk of pregnancy. The number of antenatal care consultations did not change for pregnant women suffering from chronic diseases or high-risk cases.

Data on study variables were extracted from the electronic health records of participants. The physician or midwife of the center was interviewed in cases where a variable was missed. Those participants with > 20% missing data were excluded from the study.

### Ethical considerations

The research purpose and methodology were subjected to scrutiny by the Research Ethics Committee of the Isfahan University of Medical Sciences, Isfahan, Iran (Code: IR.MUI.MED.REC.1400.367).

### Statistical analysis

Categorical variables were described as frequencies (%) and continuous as Mean ± SD. After assessing normality distribution- with the Shapiro-Wilk test- the Mann-Whitney test was used to compare the mean of continuous characteristics between pre-pandemic (2019) and intra-pandemic (2020) periods. Chi-square and Fisher's exact tests were used. A 2-sided p-value < 0.05 was considered statistically significant. The data were analyzed using Statistical Package for the Social Sciences (SPSS), version 22.0.

## 3. Results

A total of 5711 cases were investigated, of which 3477 (61%) were referred in 2019 (nonexposed) and 2234 (39%) during the COVID-19 pandemic in 2020 (exposed). As shown in table I, the mean age of pregnant women was significantly higher in exposed group (32 ± 5 yr) in comparison to nonexposed group (29 ± 7 yr); (p < 0.0001). Also, the mean gravidity (1.3 ± 1.6) and the mean interpregnancy interval (5.6 ± 4.0) were significantly shorter during the COVID-19 pandemic in comparison to nonexposed group (p < 0.001).

Although the number of cases with a history of abortion (25%; n = 548) was significantly lower during the COVID-19 pandemic than nonexposed; however, the percentage of people with a previous history of preeclampsia was higher in this period (4.6%; n = 102).

Generally, the number of mothers with a history of the underlying disease who referred to a comprehensive healthcare center during the COVID-19 pandemic (47%; n = 1052) was lower about 6% in comparison to nonexposed group (41%; n = 1414) (p < 0.001). Also, diabetes was more prevalent among mothers who referred to a comprehensive healthcare center during the COVID-19 pandemic about 1% (p < 0.001, Table I). As shown in table II, the prevalence of abortion (2%; n = 48) and abdominal or flank pain (3%; n = 58) higher by 2x in exposed group compared to nonexposed group, which was statistically significant. Also, in exposed group, 16% (n = 362) of pregnant women had vaginal delivery, which higher about 4% in comparison to nonexposed group (p < 0.001).

Regarding age, 24% (n = 544) of pregnant women were categorized as high risk during the COVID-19 pandemic. This rate was 21% (n = 738) in nonexposed group, indicating a significant increase of 3% (p < 0.001).

In exposed group, the prevalence of hypertension and gestational diabetes mellitus was 5% (n = 109) and 20% (n = 445), which were higher about 2% and 4%, respectively, compared to nonexposed group. NMR was also lower about 5% during the COVID-19 pandemic compared to nonexposed group, which was statistically significant. As shown in table II, the COVID-19 pandemic imposed no significant effect on mothers' and neonates' other characteristics compared to nonexposed group.

**Table 1:** Demographic and baseline maternal characteristics of pregnant women complications in theexposed group and non-exposed group


**Characteristics **		**Total (n = 5711)**	**Nonexposed group (n = 3477)**	**Exposed group (n = 2234)**	**P-value**
Residence*					
	**> 20,000 inhabitants**	2525 (44.2)	1564 (45)	961 (43)	
	**< 20,000 inhabitants**	1242 (21.7)	707 (20.3)	535 (23.9)	
	**Suburbs**	734 (12.9)	467 (13.4)	267 (12)	
	**Village**	957 (16.8)	564 (16.2)	393 (17.6)	0.005 ${}^{\text{b}}$
Maternal age (Yr)**		30.2 ± 6.53	28.9 ± 6.82	32.4 ± 5.30		< 0.001 ${}^{\text{a}}$
Maternal weight (kg)**		65.8 ± 12.75	65.6 ± 12.79	66.0 ± 12.69		0.132 ${}^{\text{a}}$
Gestational age (wk)**		38.1 ± 2.16	38.1 ± 2.08	38.2 ± 2.28		0.482 ${}^{\text{a}}$
Gravidity (number)**		1.6 ± 1.77	1.79 ± 1.86	1.3 ± 1.60	< 0.001 ${}^{\text{a}}$
Previous abortions*		1684 (29.5)	1136 (32.7)	548 (24.5)	< 0.001 ${}^{\text{b}}$
Pregnancy interval (Yr)**		6.9 ± 5.41	7.7 ± 6.00	5.6 ± 4.00	< 0.001 ${}^{\text{a}}$
History of preeclampsia*		220 (3.9)	118 (3.4)	102 (4.6)	0.025 ${}^{\text{b}}$
Underlying disease*		2466 (43.2)	1414 (40.7)	1052 (47.1)	< 0.001 ${}^{\text{b}}$
Digestive diseases*		128 (2.2)	70 (2)	58 (2.6)	0.146 ${}^{\text{b}}$
Kidney diseases*		101 (1.8)	61 (1.8)	40 (1.8)	0.919 ${}^{\text{b}}$
Connective tissue diseases*		19 (0.3)	11 (0.3)	8 (0.4)	0.789 ${}^{\text{b}}$
Thyroid diseases*		1238 (21.7)	743 (21.4)	495 (22.2)	0.480 ${}^{\text{b}}$
Thalassemia minor*		354 (6.2)	199 (5.7)	155 (6.9)	0.063 ${}^{\text{b}}$
Chronic hypertension*		63 (1.1)	35 (1.0)	28 (1.3)	0.384 ${}^{\text{b}}$
Heart disease*		59 (1.0)	30 (0.9)	29 (1.3)	0.112 ${}^{\text{b}}$
Asthma*		50 (0.9)	26 (0.7)	24 (1.1)	0.196 ${}^{\text{b}}$
Coagulation disorder*		6 (0.1)	5 (0.1)	1 (0.04)	0.414 ${}^{\text{c}}$
Genital malformations*		13 (0.2)	5 (0.1)	8 (0.4)	0.097 ${}^{\text{b}}$
Diabetes*		143 (2.5)	70 (2)	73 (3.3)	0.003 ${}^{\text{b}}$
History of breast cancer*		3 (0.1)	2 (0.1)	1 (0.04)	0.999 ${}^{\text{c}}$
Hepatitis*		18 (0.3)	8 (0.2)	10 (0.4)	0.152 ${}^{\text{b}}$
Psychiatric disorder*		136 (2.4)	76 (2.2)	60 (2.7)	0.227 ${}^{\text{b}}$
Multiple sclerosis*		20 (0.4)	10 (0.3)	10 (0.4)	0.318 ${}^{\text{b}}$
Iron deficiency anemia*		137 (2.4)	84 (2.4)	53 (2.4)	0.917 ${}^{\text{b}}$
Sickle anemia*		5 (0.1)	3 (0.1)	2 (0.1)	0.999 ${}^{\text{c}}$
Thrombophilia*		13 (0.2)	5 (0.1)	8 (0.4)	0.07 ${}^{\text{b}}$
Epilepsy*		48 (0.8)	28 (0.8)	20 (0.9)	0.716 ${}^{\text{b}}$
Other diseases*		344 (6)	181 (5.2)	173 (7.3)	0.001 ${}^{\text{b}}$
*Data presented as n (%). **Data presented as Mean ± SD. ${}^{\text{a}}$ Mann-Whitney test, ${}^{\text{b}}$ Chi-square test, ${}^{\text{c}}$ Fisher exact test. Because of the missing value, sum of percentage may not be equal to 100%. Median (Q1, Q3) of Gravidity number, total, pre-pandemic, and intra-pandemic period were the same = 1 (0, 2)					

**Table 2:** Comparisons of maternal, neonatal characteristics, and pregnancy complications in the pre- and intra- COVID-19 pandemic periods


**Characteristics**	**Total (n = 5711)**	**Nonexposure group (n = 3477)**	**Exposure group (n = 2234)**	**P-value**
Abortion*	87 (1.5)	39 (1.1)	48 (2.1)	0.002 ${}^{\text{a}}$
Vaginal delivery*	766 (13.4)	404 (11.6)	362 (16.2)	< 0.001 ${}^{\text{a}}$
High-risk pregnancy*				
**Maternal age < 18 and** **> 35 yr **	3235 (56.6)	738 (21.2)	544 (24.3)	< 0.001 ${}^{\text{a}}$
**Pregnancy interval < 2 yr **	494 (8.6)	264 (4.6)	230 (10.3)	< 0.001 ${}^{\text{a}}$
**Gravidity > 5 times **	397 (7)	282 (8.1)	115 (5.1)	< 0.001 ${}^{\text{a}}$
Danger signs during pregnancy*				
**Preeclampsia**	379 (6.6)	225 (6.5)	154 (6.9)	0.531 ${}^{\text{a}}$
**Hypertension **	221 (3.9)	112 (3.2)	109 (4.9)	0.002 ${}^{\text{a}}$
**Diabetes **	990 (17.3)	545 (15.7)	445 (19.9)	< 0.001 ${}^{\text{a}}$
**Fever **	52 (0.9)	30 (0.9)	22 (1)	0.636 ${}^{\text{a}}$
**Thromboembolism **	89 (1.6)	46 (1.3)	43 (1.9)	0.073 ${}^{\text{a}}$
**Headache **	294 (5.1)	193 (5.6)	101 (4.5)	0.086 ${}^{\text{a}}$
**Leakage **	15 (0.3)	9 (0.3)	6 (0.3)	0.944 ${}^{\text{a}}$
**Heartbeat **	66 (1.2)	43 (1.2)	23 (1)	0.475 ${}^{\text{a}}$
**Spotting bleeding **	135 (2.4)	88 (2.5)	47 (2.1)	0.300 ${}^{\text{a}}$
Complications of postpartum*				
**Dizziness **	51 (0.9)	36 (1)	15 (0.7)	0.154 ${}^{\text{a}}$
**Hemorrhage **	461 (8.1)	284 (8.2)	177 (7.9)	0.740 ${}^{\text{a}}$
**Breath shortness**	3678 (64.4)	2267 (65.2)	1411 (63.2)	0.116 ${}^{\text{a}}$
**Abdominal/flank pain**	111 (1.9)	53 (1.5)	58 (2.6)	0.004 ${}^{\text{a}}$
**Orthopedics**	3678 (64.4)	2267 (65.2)	1411 (63.2)	0.116 ${}^{\text{a}}$
**Unexplained cough**	3678 (64.4)	2267 (65.2)	1411 (63.2)	0.116 ${}^{\text{a}}$
**Inferior limb edema **	3678 (64.4)	2267 (65.2)	1411 (63.2)	0.116 ${}^{\text{a}}$
**Genitourinary problems **	79 (1.4)	53 (1.5)	26 (1.2)	0.255 ${}^{\text{a}}$
**Neonatal characteristics**				
Birth death*	36 (0.6)	29 (0.8)	7 (0.3)	0.016 ${}^{\text{a}}$
Birth weight (gr)**	3065 ± 541	3068.4 ± 550.21	3058.5 ± 526.7	0.488 ${}^{\text{b}}$
Birth height (cm)**	49 ± 4	49.4 ± 3.87	49.3 ± 3.61	0.048 ${}^{\text{b}}$
Birth head size (cm)**	34.6 ± 2.8	34.6 ± 2.88	34.6 ± 2.62	0.082 ${}^{\text{b}}$
*Data presented as n (%). **Data presented as Mean ± SD. a: Chi-square test, b: Mann-Whitney test. Because of missing value, the sum of percentage may not be equal to 100%				

## 4. Discussion

This study aimed to investigate the impact of the COVID-19 pandemic on maternal and neonatal health by comparing 2 periods from March-May 2019, and the same period in 2020. According to the findings, the total number of pregnancies declined in 2020 (22,343 cases) compared to 2019 (3477 cases). In 2020 (in comparison to 2019), the mean age of pregnant women was higher, the interpregnancy interval was shorter, the number of previous pregnancies was lower, the percentage of abortion history was lower, the history of preeclampsia was higher, and diabetes was more prevalent. Also, abortion and vaginal delivery were more prevalent in 2020 in comparison to 2019. The frequency of hypertension and diabetes during pregnancy was higher in 2019 compared to 2020, and the frequency of stillbirth was lower in 2020. There was no difference between the 2 periods concerning preterm labor and maternal weight gain. There was no difference between the pregnancy complications periods except for flank pain.

A study performed in Tehran reported that the mean age of pregnant women was significantly lower during the COVID-19 pandemic than nonexposed group. The number of previous pregnancies and the number of alive neonates were higher during the COVID-19 pandemic than before (9).

There was no significant difference in pregnancy complications (including preeclampsia, hypertension, and gestational diabetes), delivery mode, and frequency of stillbirth before and during the COVID-19 pandemic. The frequency of preterm birth and LBE was significantly declined during the COVID-19 pandemic compared to nonexposed group. In addition, the weight of neonates was higher during the COVID-19 pandemic than nonexposed group.

By a systematic review, which intended to evaluate the effect of COVID-19 on the maternal, fetus, and neonate consequences, reported higher rates of stillbirth and maternal mortality rate during the COVID-19 pandemic. No significant effect was observed for other outcomes, including gestational diabetes, gestational hypertension disorders, induction of labor, delivery mode (spontaneous vaginal delivery, cesarean section (C-section), or instrumental delivery), postpartum hemorrhage, neonate death, low-birth weight ( < 2500 gr), and preterm delivery rate (7). A retrospective cohort study that intended to investigate the effect of the first COVID-19 peak on prenatal morbidity and mortality found a higher rate of stillbirth during the COVID-19 pandemic than nonexposed group. Gestational age, preterm labor rate, pregnancy complications, and complications of delivery and its type were similar in both groups (10). The other study in line with the present study's findings showed no association between preterm labor and stillbirth and birth during the COVID-19 pandemic (11). In a study conducted in Nigeria, the authors mentioned a 22% increase in stillbirth and 23% in neonatal mortality rate during the COVID-19 pandemic compared to nonexposed group (12). The difference between the findings of the study conducted in Nigeria and the present study can be attributed to differences in participants' social, cultural, and economic characteristics and the retrospective design of the Nigeria study.

In a study conducted in London, reported lower rates of pregnancy hypertension and higher rates of stillbirth among pregnant women during the COVID-19 pandemic compared to nonexposed group, which is against the present study's findings. However, there is no difference between these 2 studies concerning the frequency of preterm labor (13). Another study reported no difference in the rate of preterm delivery, stillbirth, and other perinatal complications (14). In another study conducted during the COVID-19 pandemic, the authors mentioned declined rate of C-sections with the observance of new protocols; however, this decline was not associated with increased mortality and morbidity. The current study's findings contained similar results regarding natural delivery rates (15). A considerable increase in natural delivery rates during the COVID-19 pandemic is also mentioned in another study (16). Another study reported a significant increase in the percentage of high-risk pregnancies, a 2.5-fold increase in maternal admission in intensive care units, and a high percentage of pregnancy complications as some significant consequences of declined maternal referral for antenatal care, which was due to lockdown and fear of contracting COVID-19 when referring to healthcare centers (17).

Although our findings indicated an increased prevalence of diabetes and hypertension, this finding did not affect stillbirth during the COVID-19 pandemic. However, it can be used to justify the higher frequency of abortion in 2020, in comparison to 2019, by affecting other confounding factors such as decreased physical activity, increased consumption of high-calorie substances, improper control of blood sugar and blood pressure, or higher prevalence of underlying diseases among various factors. Older age of pregnant women, a history of preeclampsia, inappropriate diet, and decreased physical activity due to lockdowns imposed during the COVID-19 pandemic probably contributed to the higher prevalence of diabetes and hypertension among pregnant women.

Regarding the reduced number of prenatal care sessions during the COVID-19 pandemic, which was applied according to the maternal-fetal-medicine guidance for COVID-19 (18), the diagnosis rate of diabetes and hypertension was higher, indicating the strength of the health system. Nevertheless, the reduced number of prenatal care sessions did not affect the C-section outcome, labor consequences, mother's weight gain, preterm labor, and indicators of weight, height, and head circumference. Therefore, based on the findings, shortage of health human resources, and increased sensitivities to quality of healthcare services, the authors recommend reducing the number of antenatal care sessions from 8 to 4 for low-risk pregnancies.

Herein, some limitations and challenges must be considered before applying the findings, including sole investigation of public healthcare centers, short study period, and lack of sufficient information about real causes of abortion.

## 5. Conclusion

While rigorous evidence is not available yet, evidence provided by this study indicated the negative impact of the COVID-19 pandemic on referring to healthcare centers to receive antenatal care among pregnant women living in cities with a population of > 20,000. A reverse trend was observed in rural areas and cities with a population of < 20,000. However, this study showed that the COVID-19 pandemic imposes no significant effect on mothers' and neonates' health.

##  Conflict of Interest

The authors declare no conﬂict of interest. 
